# Mutual Effects of Hydrogen Bonding and Polymer Hydrophobicity on Ibuprofen Crystal Inhibition in Solid Dispersions with Poly(*N*-vinyl pyrrolidone) and Poly(2-oxazolines)

**DOI:** 10.3390/pharmaceutics13050659

**Published:** 2021-05-04

**Authors:** Xiaoning Shan, Maryam A. Moghul, Adrian C. Williams, Vitaliy V. Khutoryanskiy

**Affiliations:** Reading School of Pharmacy, University of Reading, Whiteknights, P.O. Box 224, Reading RG6 6AD, UK; Xiaoning.Shan@pgr.reading.ac.uk (X.S.); mmoghul.01@gmail.com (M.A.M.); a.c.williams@reading.ac.uk (A.C.W.)

**Keywords:** solid dispersions, hydrogen bonding, hydrophobicity, poly(*N*-vinyl pyrrolidone), poly(2-oxazolines), crystallinity, hydrophobic drug, amorphous, ibuprofen

## Abstract

Poly(*N*-vinyl pyrrolidone) (PVP), poly(2-methyl-2-oxazoline) (PMOZ), poly(2-ethyl-2-oxazoline) (PEOZ), poly(2-*n*-propyl-2-oxazoline) (PnPOZ), and poly(2-isopropyl-2-oxazoline) (PiPOZ) were used to prepare solid dispersions with ibuprofen (IB), a model poorly-water soluble drug. Dispersions, prepared by solvent evaporation, were investigated using powder X-ray diffractometry, differential scanning calorimetry, and FTIR spectroscopy; hydrogen bonds formed between IB and all polymers in solid dispersions. PMOZ, the most hydrophilic polymer, showed the poorest ability to reduce or inhibit the crystallinity of IB. In contrast, the more hydrophobic polymers PVP, PEOZ, PnPOZ, and PiPOZ provided greater but similar abilities to reduce IB crystallinity, despite the differing polymer hydrophobicity and that PiPOZ is semi-crystalline. These results indicate that crystallinity disruption is predominantly due to hydrogen bonding between the drug molecules and the polymer. However, carrier properties affected drug dissolution, where PnPOZ exhibited lower critical solution temperature that inhibited the release of IB, whereas drug release from other systems was consistent with the degree of ibuprofen crystallinity within the dispersions.

## 1. Introduction

Whilst oral delivery remains the most common route for drug administration, most new active pharmaceutical ingredients are poorly water soluble and thus not well-absorbed after oral administration. Solid dispersion, defined as the dispersion of one or more active ingredient in a carrier or matrix at solid state, is an established platform technology to enhance the dissolution rate and improve the apparent solubility of a drug and, hence, increase the bioavailability of a range of poorly water soluble drugs [1,2,3]. Several classes of hydrophilic polymers have been used as carriers to prepare solid dispersions, including PVP [4,5,6] and its derivatives [7,8,9], polyethylene glycols [10,11], cellulose ethers [12,13] and poloxamers [14,15].

In solid dispersions, polymer–drug interactions can provide stability to the system by restricting the mobility of the drug molecules in the polymer matrix. Common interactions between drugs and polymers include ionic, hydrophobic, dipole–dipole, Van der Waals, and hydrogen bonding [16,17,18]. Hydrogen bonding is typically detected between drugs and polymers in solid dispersions, as reported extensively, for example, between IB and PVP [19,20], esomeprazole and hydroxypropyl methylcellulose (HPMC) [21], flurbiprofen and poly(ethylene oxide) [22], and for nifedipine and Eudragit^®^ [23], indicating that this is a key mechanism in the successful formation of amorphous or semi-crystalline solid dispersions. In contrast, there are relatively few studies exploring the effects of carrier hydrophobicity on crystallization inhibition [16,24,25]. However, research typically focuses on hydrogen bonding or hydrophobicity, with little consideration given to the mutual effects of hydrogen bonding and polymer hydrophobicity on drug crystal inhibition.

Poly(2-oxazolines) have been reported as an alternative to PVP in solid dispersions for solubility and dissolution rate enhancement of poorly-water soluble drugs. For example, Fael et al. [26] found that a lower molecular weight of PEOZ (5000 g/mol) was superior to a higher molecular weight of the polymer (50,000 g/mol) in improving the dissolution behavior of glipizide. Boel et al. [27] showed that PEOZ maintained supersaturation of itraconazole and fenofibrate to a similar extent as PVP, poly(vinylpyrrolidone-*co*-vinyl acetate) (PVP-VA), and HPMC. Everaerts et al. [28] selected PEOZ, PnPOZ, poly(2-sec-butyl-2-oxazoline) (PsecBuOZ), and a combination of PEOZ with either PnPOZ or PsecBuOZ as carriers for amorphous solid dispersions with six drugs, and highlighted the potential of poly(2-oxazolines) as a novel polymer carrier to form amorphous solid dispersions.

In our previous work [29], PVP and a series of water-soluble poly(2-oxazolines) including PMOZ, PEOZ, PnPOZ, and PiPOZ were used to prepare solid dispersions with haloperidol. The effects of polymer hydrophobicity and their semi-crystalline nature on drug crystallinity were demonstrated. However, hydrogen bonding between haloperidol and poly(2-oxazolines) was almost absent due to the poor hydrogen bond donating ability of the haloperidol hydroxyl group.

In order to explore the impacts of both polymer hydrophobicity and drug–polymer hydrogen bonding, we selected IB, a hydrophobic crystalline drug and strong hydrogen bond donor (because of its carboxylic group), to prepare solid dispersions with poly(2-oxazolines) and PVP. Dispersions were prepared by solvent evaporation and characterized using FTIR spectroscopy, differential scanning calorimetry, and powder X-ray diffractometry. Solubility parameters and Flory–Huggins interaction parameters were calculated to predict drug–polymer miscibility, and drug dissolution studies were conducted to further explore the relationship between IB crystallization inhibition and release from the dispersions.

## 2. Materials and Methods

### 2.1. Materials

Poly(2-ethyl-2-oxazoline) (PEOZ), 50 kDa (polydispersity index, PDI 3-4); poly(*N*-vinyl pyrrolidone) (PVP), 55 kDa (K-value 30); and buffer tablets, pH 6.8 were from Sigma-Aldrich (Gillingham, UK). Poly(2-methyl-2-oxazoline) (PMOZ), poly(2-n-propyl-2-oxazoline) (PnPOZ), and poly(2-isopropyl-2-oxazoline) (PiPOZ) were synthesized according to our previously reported procedure [29]. Ibuprofen (IB) was from Tokyo Chemistry Industry (Japan). *N, N*-dimethylacetamide (DMA) was from Fisher Scientific (Loughborough, UK).

### 2.2. Preparation of Polymer–IB Solid Dispersions

Solid dispersions of polymer–IB were prepared in different repeating unit/drug molar ratios by solvent evaporation. DMA (1 mL) was used to dissolve 25 mg of IB with varying amounts of each polymer depending on the repeating unit/drug molar ratios. After dissolution, the solution was transferred to a Petri dish and the solvent was removed by evaporation at 50 °C on a heating base. The resultant solid was kept under vacuum for 72–96 h to remove residual DMA.

### 2.3. Characterization of Solid Dispersions

#### 2.3.1. Powder X-ray Diffractometry (PXRD)

A small amount of each dry sample (~20 mg) was placed on a silica plate and analyzed in a Bruker D8 ADVANCE PXRD using Cu Kα radiation (λ = 1.5406 Å) over 5–64° for 1 h, with a step of 0.05°(2θ) and count time of 1.2 s at 40 mV, 40 mA, with the sample rotated at 30 rpm. The results were analyzed using EVA software and the background for each sample was removed.

#### 2.3.2. Differential Scanning Calorimetry (DSC)

Thermal analysis of pure drug, polymers, and solid dispersions was performed using DSC (TA Instruments). Samples (3–5 mg) were loaded into pierced T_zero_ aluminum pans. The thermal behavior of each sample was investigated in a nitrogen atmosphere from 10 to a maximum of 220 °C at 10 °C/min. The degree of sample crystallinity was determined by the specific enthalpy (ΔΗ) of the drug melting peak using TA universal analysis software and was calculated as the ratio of ΔΗ of the drug in the solid dispersions to ΔΗ of pure IB (taken as 100% crystalline). Since the drug content in the dispersion was only a fraction of the sample weight, the degree of crystallinity was normalized according to the following equation:

(1) $$\mathrm{Crystallinity}\;(\%)=\left(\Delta H_{s}\;\times \frac{W_{s}}{W_{i}}\right)/\Delta H_{i}\;\times 100$$

where ΔΗ_s_ is the ΔΗ of the drug in the solid dispersion, melting around 76 °C (melting point of IB), ΔΗ_i_ is the ΔΗ of pure IB, W_s_ is the weight of solid dispersions, and W_i_ is the weight of IB in solid dispersions.

#### 2.3.3. Fourier Transform Infrared (FTIR) Spectroscopy

FTIR spectra were recorded on a Nicolet iS5 spectrometer using a diamond ATR (attenuated total reflection) accessory. After a background scan was collected, samples were placed on the crystal and scanned from 4000–400 cm^−1^ at a resolution of 4 cm^−1^ and with an average of 64 scans. OMINIC software was used for spectral analysis.

### 2.4. In Vitro Dissolution Studies

Dissolution of IB from solid dispersions ([polymer repeat unit]/[drug] = 1:1 mol/mol) used USP Apparatus II (paddle method) at 37 ± 0.5 °C with paddles at 50 rpm and simulated intestinal fluid (SIF) (PBS, pH = 6.8). A pharmaceutical grade empty vegan clear capsule size “0” filled with solid dispersion (equivalent to 100 mg drug) was placed in 900 mL SIF with a sinker. Samples (5 mL) were withdrawn at 2, 5, 10, 20, 40, 60, 80, 100, and 120 min, and filtered using a 0.45-μm syringe filter; an equal volume of SIF was added to the dissolution medium to maintain the volume. The drug was assayed by UV-visible spectroscopy at 265 nm. All dissolution studies were performed in triplicate.

### 2.5. Statistical Analysis

All solid dispersions for each polymer at all drug loadings were prepared three times independently. All analyses, PXRD, DSC, FTIR, and dissolution studies were in triplicate. Data are expressed as mean ± standard deviation.

## 3. Results and Discussion

### 3.1. Preparation and Characterization of Solid Dispersions

**Figure 1:**
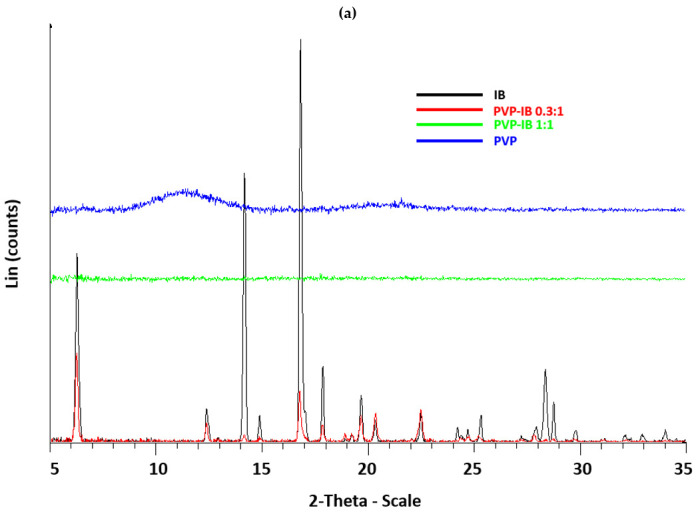

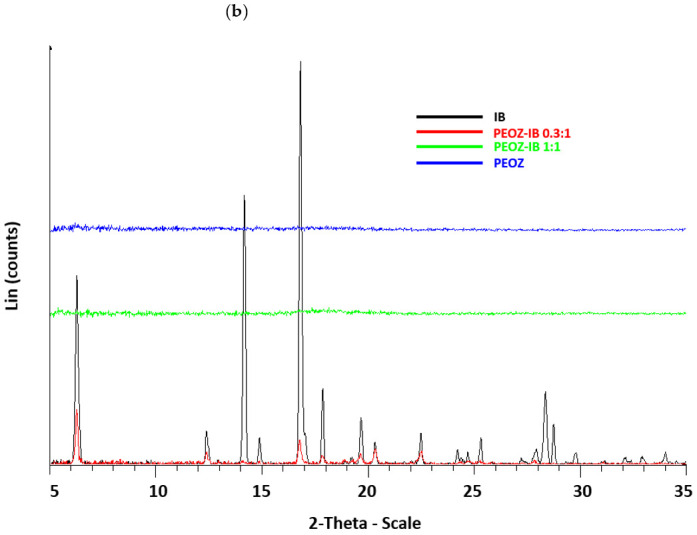

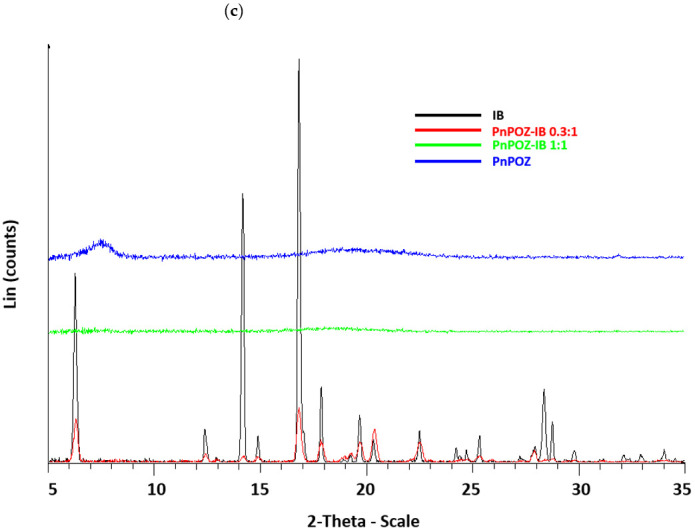

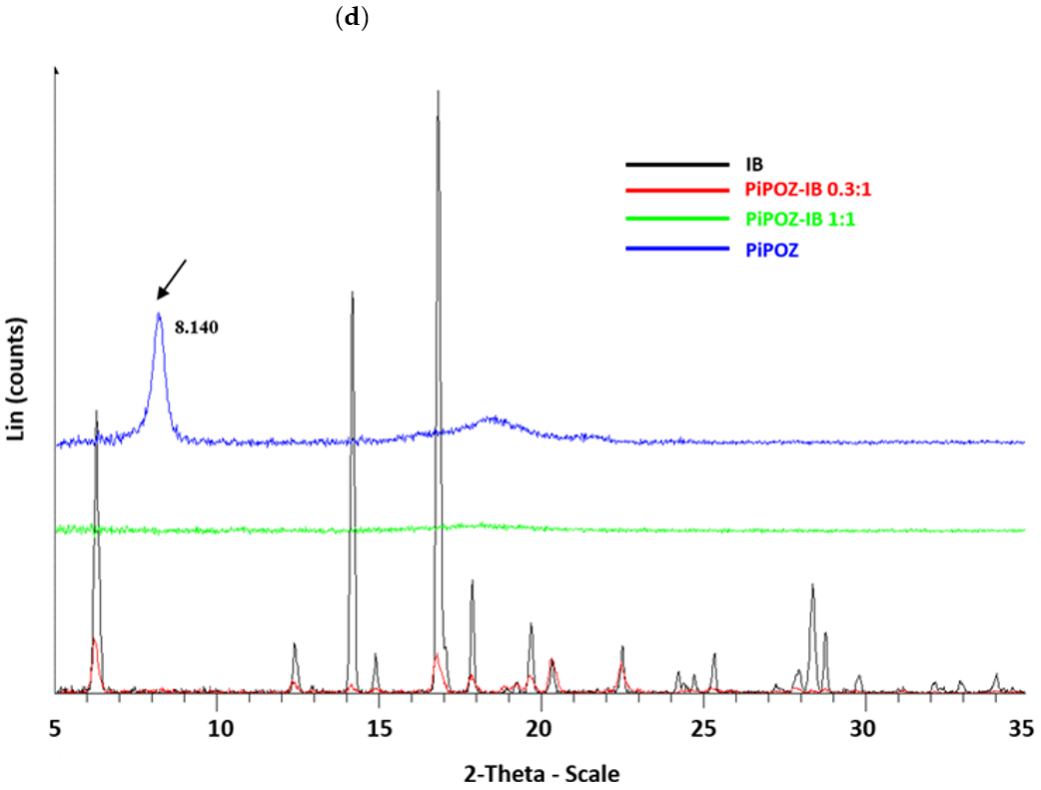

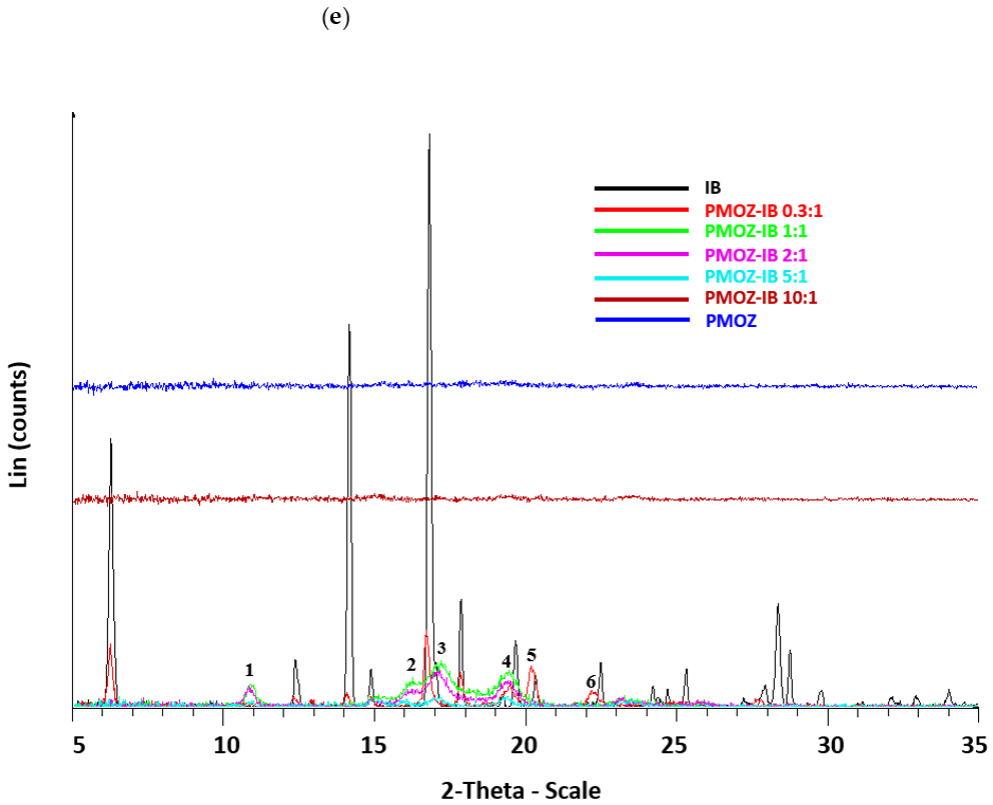
X-ray diffraction diagrams of PVP-IB SDs (**a**), PEOZ-IB SDs (**b**), PnPOZ-IB SDs (**c**), PiPOZ-IB SDs (**d**), and PMOZ-IB SDs (**e**).

To evaluate the effects of different polymers on the crystallinity of IB, solid dispersions were prepared by solvent evaporation and were characterized by DSC, PXRD, and FTIR, with DSC used to calculate the crystallinity of IB in the dispersions. The X-ray diffractogram of IB (Figure 1) shows multiple distinctive peaks, notably at 6.2°, 12.3°, 14.1°, 14.9°, 16.8°, 17.9°, 19.6°, 20.3°, 22.5°, 25.3°, 28.4°, and 28.8°, in agreement with the literature [30] and demonstrating the crystalline nature of pure IB.

Clearly, the drug is diluted when included in the polymer dispersion, and so peak intensities fall in all solid dispersions with PVP, PEOZ, PnPOZ, and PiPOZ at molar ratio [polymer]/[IB] = 0.3:1. In these systems, the drug is in excess of the polymer hydrogen bond acceptor repeat units and so is expected to remain largely crystalline. However, when the molar ratio of polymer repeat unit to IB is 1:1, the X-ray data in Figure 1 indicate that IB crystallinity is completely lost (although PMOZ systems showed some anomalous results, discussed below). It is also interesting to note that PiPOZ alone is semi-crystalline and presents a feature at 8.14° 2θ, but this is lost at both 0.3:1 (where IB is in excess) and at a 1:1 mole ratio with the drug (highlighted in Figure 1d). This demonstrates that whilst solid dispersion studies typically focus on disruption of drug crystallinity, clearly the interactions between drug and carrier can also affect the nature and properties of the polymeric dispersant.

When dispersed in PMOZ, the X-ray data indicate different behavior for ibuprofen. As above, at a 0.3:1 molar ratio, where the drug is in excess, there is a reduction in the intensity of the peaks from ibuprofen due to the dilution effect. Some peaks observed in samples at this ratio were slightly shifted from their positions seen with pure ibuprofen at 19.6°, 20.3°, and 22.5° (denoted as 4, 5, and 6, respectively, in Figure 1e) while the characteristic peak of IB at 16.8° 2θ did not significantly move, indicating that IB was predominantly in its original form. However, in contrast with the other polymers, it is clear that ibuprofen has some structure in PMOZ at 1:1, 1:2, and 1:5 drug:polymer compositions, with a series of broader diffraction features seen between 16 and 20°; again, these features show reduced intensities as the drug is further diluted in the polymer. For these systems, the original strong diffraction peak from the initial crystalline ibuprofen at 16.8° 2θ is lost, and broader features at 16.1°, 17.2°, and 19.5° 2θ are seen (peaks 2, 3, and 4, respectively, in Figure 1e). Further, a new feature at 10.5° is seen in these systems, which is absent from the pure ibuprofen diffractogram (peak 1). The changes to the diffraction peak positions and breadth of the new features are consistent with a semi-crystalline structure, but one that differs from the initial ibuprofen crystal lattice. Racemic ibuprofen is known to be polymorphic, but the diffraction peaks seen for the drug when dispersed in PMOZ are not consistent with the reported form II polymorph [31]. Thus, in our system, it is feasible that a semi-crystalline PMOZ-IB complex is formed. Finally, at a 1:10 drug:polymer composition, all diffraction peaks were lost, suggesting the formation of an amorphous dispersion.

FTIR spectra were recorded from the components and dispersions to probe molecular interactions. Functional groups of particular interest are the carboxyl group of IB [−C=O(OH)], where the −OH acts as a proton donor, and the carbonyl group (–C=O) and the nitrogen atom of polymers, which act as proton acceptors. In addition, correlation between frequency shifts and intermolecular interaction between drugs and polymers in solid dispersions is well known [32,33,34], and so was used to investigate hydrogen bonding in our polymer–drug solid dispersion systems.

**Figure 2:**
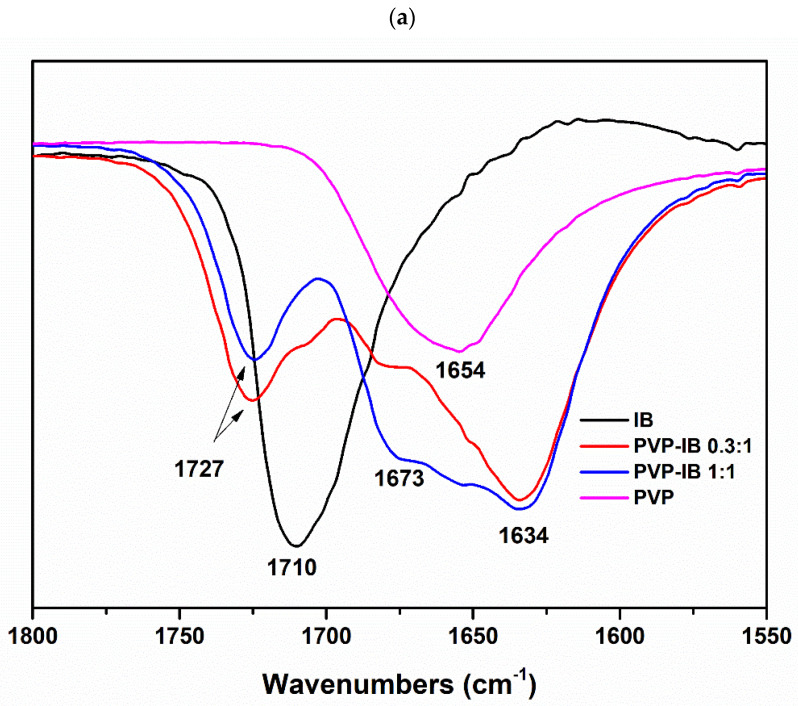

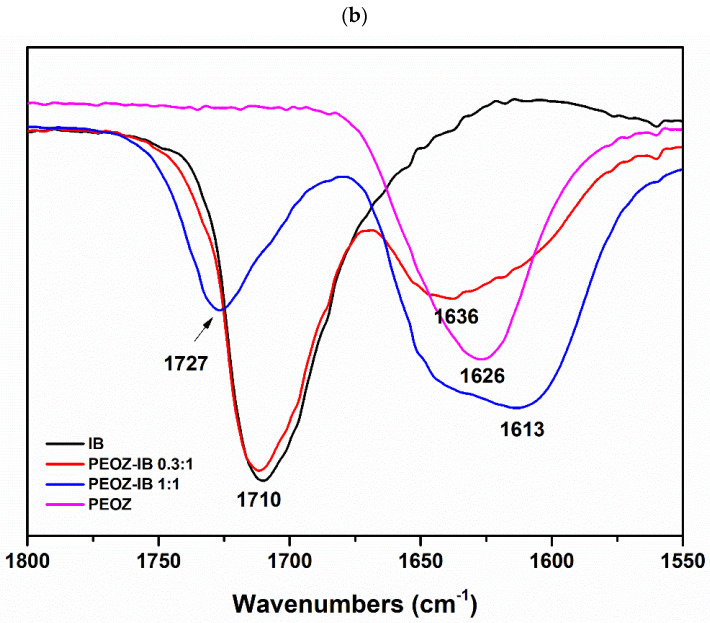

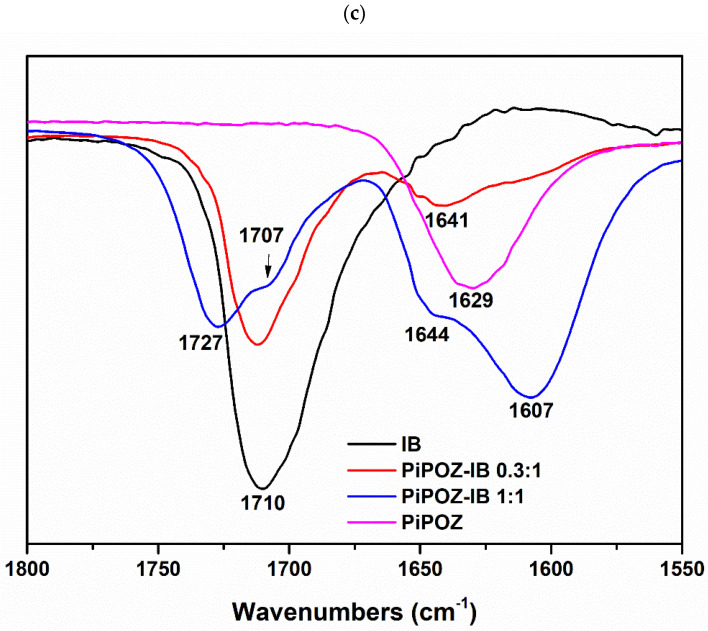

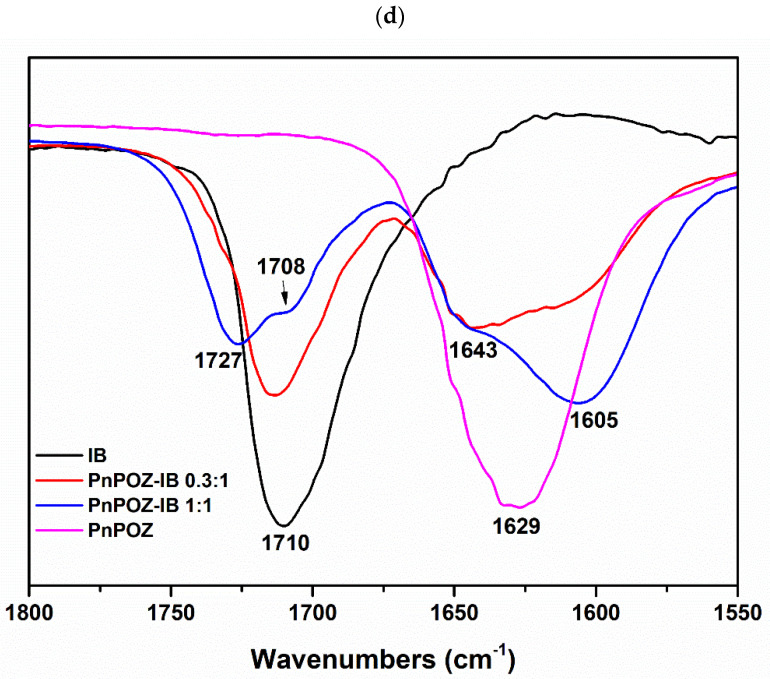

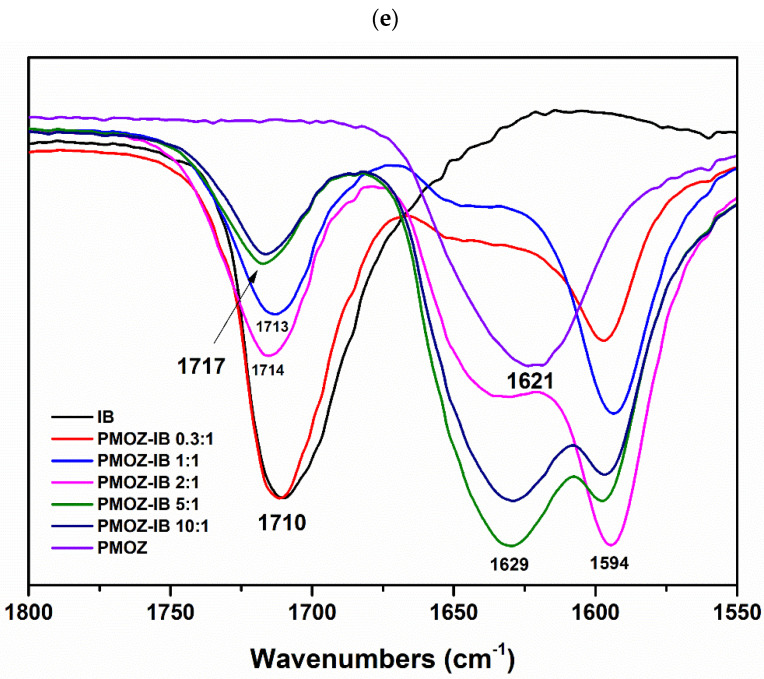
FTIR spectra of PVP-IB SDs (**a**), PEOZ-IB SDs (**b**), PnPOZ-IB SDs (**c**), PiPOZ-IB SDs (**d**), and PMOZ-IB SDs (**e**) in the range of 1800–1550 cm^−1^.

The FTIR spectra of IB, polymers, and polymer–IB solid dispersions are shown in Figures S1 and S2. For clarity, the spectra are expanded between 1800–1550 cm^−1^ in Figure 2. IB vibrational frequencies and their assignments are given in Table S1 and agree with the literature [35]. Briefly, absorption bands between 3100–2800 cm^−1^ are attributed to C−H stretching modes, with peak intensities gradually reducing as the drug quantity falls in the solid dispersions (Figure S2). Two medium intensity features, appearing at 2725 cm^−1^ and 2633 cm^−1^ in the spectrum of IB, can be assigned to the stretching vibration of the cyclic dimerized hydroxyl groups, which is subjected to intermolecular hydrogen bonding [19,36] (Figure S1). However, these bands are lost in the spectra of amorphous solid dispersions, indicating that the drug dimeric structure is lost as a result of interaction with the polymers.

The FTIR spectrum of IB shows a strong carbonyl stretching mode at 1710 cm^−1^ (Figure 2), which shifted to higher wavenumbers when dispersed in the polymers, and especially at ratios where X-ray diffraction showed no drug crystallinity (i.e., 1:1 ratios). These red shifts are summarized in Table 1. In contrast to the polyoxazoline carriers, the spectra of 0.3:1 mol PVP:IB showed that the IB carbonyl stretching mode shifted from 1710 cm^−1^ to 1727 cm^−1^, despite the excess of IB to polymer monomer units, indicating the strong hydrogen bonding between IB with PVP may have consequential disruption to the IB crystal lattice. Furthermore, the carbonyl stretching mode for PVP at 1654 cm^−1^ was replaced by two peaks at 1634 cm^−1^ and 1673 cm^−1^, with this latter peak strengthening at [PVP]/[IB] = 1:1 (Figure 2a).

The 1:1 ratio of IB in dispersions with PEOZ, PnPOZ, and PiPOZ showed similar infrared spectra, in agreement with that for PVP. The carbonyl stretching mode of IB showed a consistent red shift of 17 cm^−1^ from 1710 cm^−1^ to 1727 cm^−1^, and the carbonyl stretch in the polymers split from the single peak at 1626 or 1629 cm^−1^ to give features at both higher and lower wavenumbers. At higher drug loadings (polymer: drug 0.3:1), the data suggest some drug–polymer interactions occurred, but these are somewhat obscured by the “free” excess IB within the systems.

As with the X-ray investigation, dispersions with PMOZ showed different molecular interactions than for the other polyoxazolines. A modest red shift in the IB carbonyl feature of 3 cm^−1^ was seen at a 1:1 stoichiometry, and when excess polymer was employed (10:1 polymer repeat unit: IB), the shift was still modest at 7 cm^−1^. The PMOZ carbonyl mode was seen at 1621 cm^−1^ in the polymer alone (Figure 2e). There is again evidence for this mode splitting in the dispersion with peaks consistently at ~1594 cm^−1^ and ~1650 cm^−1^ in the samples at 0.3:1 and 1:1 mole ratios. This peak apparently moves towards ~1629 cm^−1^ as the polymer content increases, but in fact is due to the increased contribution of the “excess” (or “free”) PMOZ carbonyl peak intensity, which overlaps and obscured the carbonyl group of PMOZ that interacts with IB. The weaker interaction of PMOZ with IB compared with that in other polyoxazoline dispersions can be attributed to PMOZ’s relatively high hydrophilicity, which inhibits its ability to disorder the hydrophobic drug molecules [29].

From the IR data, there is no evidence for hydrogen bonding between the carboxylic groups of IB and nitrogen atoms in the polymers, given the invariant C-N stretching mode (Figure S3). Although PVP can form hydrogen bonds either through the nitrogen or carbonyl group [37], steric hindrance constrains the involvement of nitrogen atom in intermolecular interactions, so the carbonyl group is more favorable for hydrogen bonding [38,39].

Overall, the changes in the carbonyl band of IB and polymers indicate a modified carbonyl environment caused by the hydrogen bonding between the carboxylic groups of IB and carbonyl groups of the polymers. The relatively high red shift of the carbonyl mode of IB at [PVP]/[IB] = 0.3:1 mol confirmed strong hydrogen bonding between the drug and PVP, and at 1:1 mole ratio, the dispersions with PVP, PEOZ, PnPOZ, and PiPOZ all showed similar red shifts of this feature, suggesting near equivalent hydrogen bond interaction strengths. In contrast, the interaction between PMOZ and IB was relatively weak as a result of PMOZ’s high hydrophilicity, but no new spectral features were found to demonstrate the presence of a novel drug: PMOZ complex.

**Figure 3:**
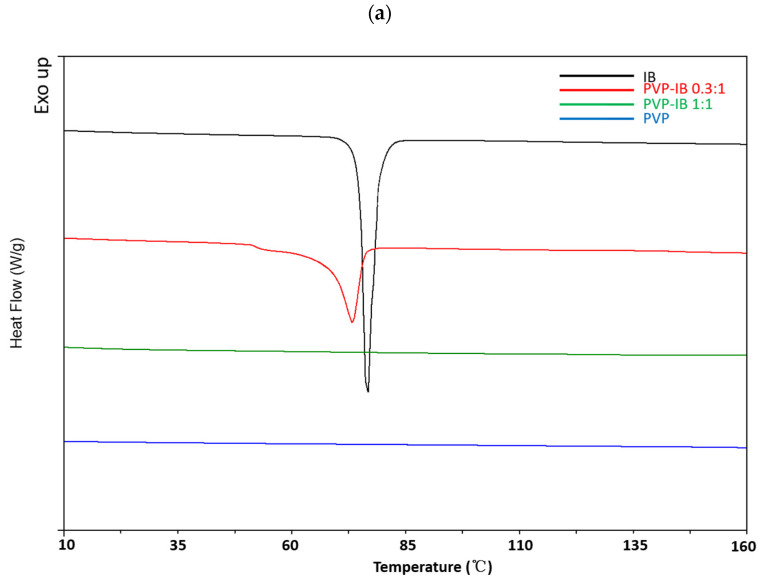

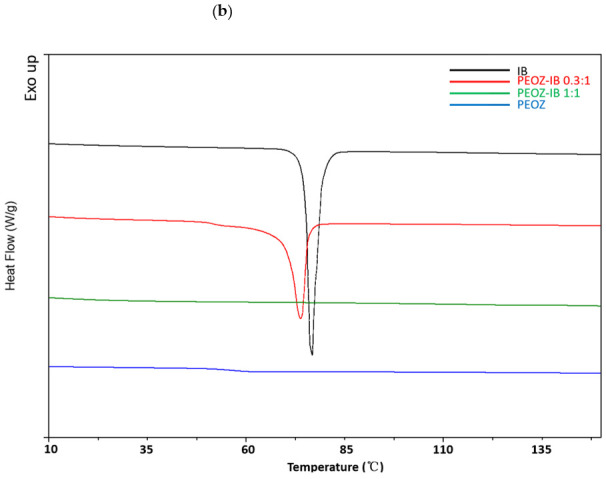

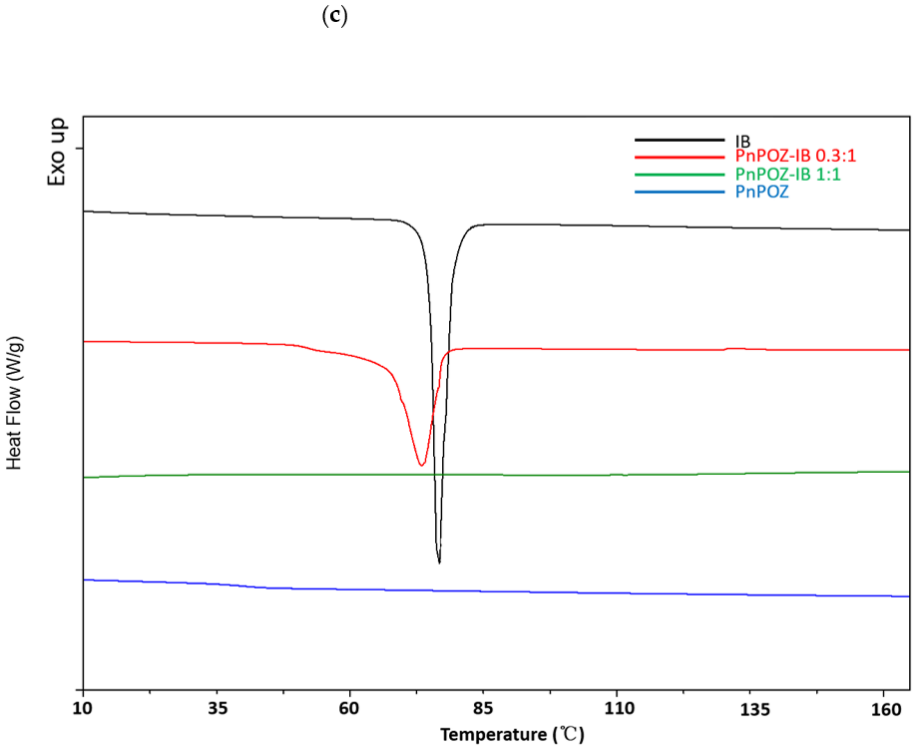

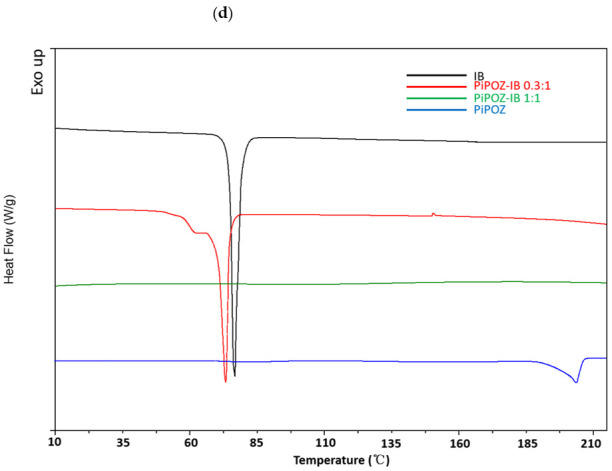

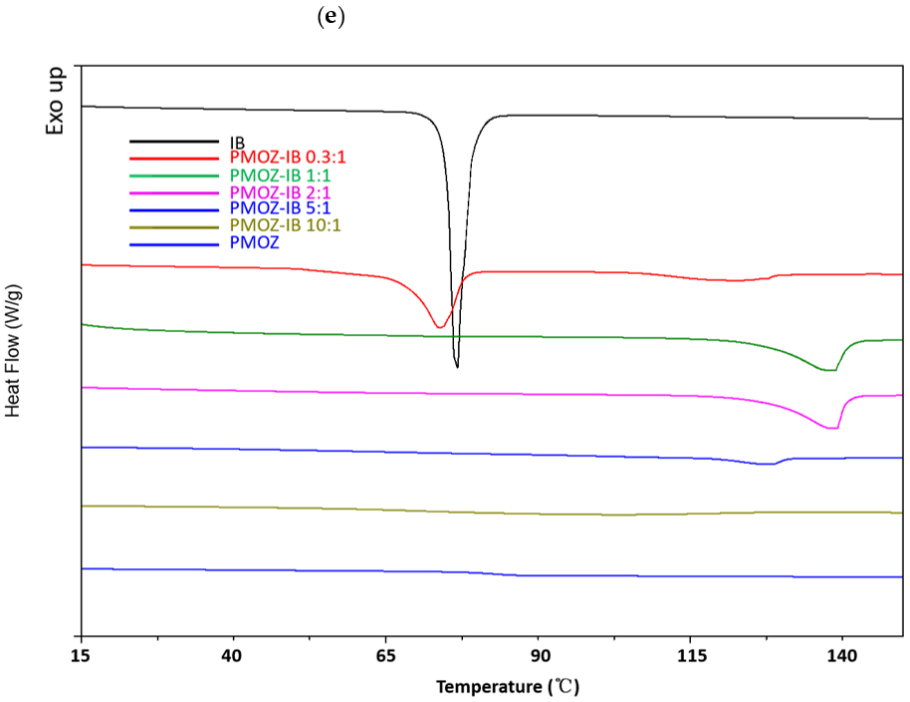
DSC thermograms of PVP-IB SDs (**a**), PEOZ-IB SDs (**b**), PnPOZ-IB SDs (**c**), PiPOZ-IB SDs (**d**), and PMOZ-IB SDs (**e**).

DSC experiments were used to investigate the thermal behavior of the solid dispersions and to estimate drug crystallinity within the dispersions. The DSC thermogram of pure IB showed a single characteristic melting peak at 76 °C, confirming its crystalline nature (Figure 3) and in agreement with previous reports [40,41]. In all dispersions at 0.3:1 mole ratio, the excess IB was seen to melt at a lower temperature, and the broadening of the melting event is consistent with disorder being introduced into the crystal lattice and interactions with the polymer occurring. At 1:1 mole ratio, the drug melting peak was lost in all dispersions except in dispersion with PMOZ, in agreement with the X-ray data.

With PMOZ, a second endothermic peak appeared at 121.2 °C in (PMOZ)/(IB) = 0.3:1, at 137.7 °C in (PMOZ)/(IB) = 1:1, at 137.9 °C in (PMOZ)/(IB) = 2:1, and at 128.3 °C in (PMOZ)/(IB) = 5:1, potentially due to semi-crystalline IB or a PMOZ-IB complex. In addition, the melting peak seen at 203.5 °C for semi-crystalline PiPOZ was lost in (PiPOZ)/(IB) solid dispersions; whilst reports tend to focus on the disruption to drug crystallinity in solid dispersions, clearly the drug also has the potential to disrupt the structure of the polymeric carrier, as indicated here.

**Figure 4:**
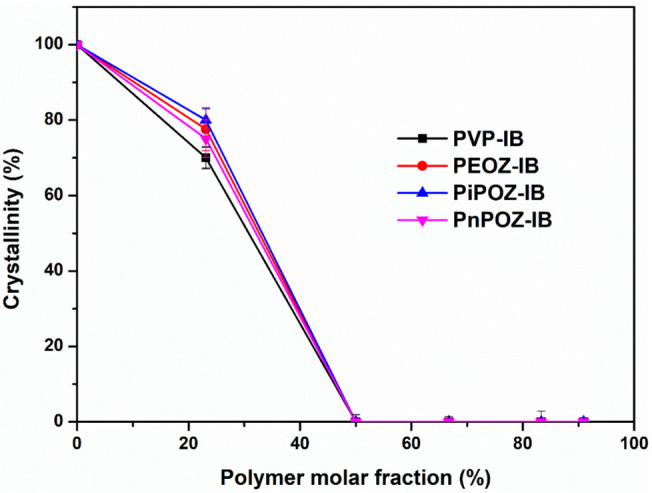
Crystallinity of polymer–IB solid dispersions as a function of polymer molar fraction.

Drug crystallinity in polymer–IB solid dispersions was calculated from the specific enthalpy of the melting peak. As can be seen from Figure 4, IB crystallinity was reduced in all dispersions with PVP, PEOZ, PnPOZ, and PiPOZ, and the drug was essentially amorphous at a molar ratio of 1:1. The crystallinity of IB in dispersions with PMOZ could not be quantified by this approach due to the formation of new thermal features and the potential formation of a complex with this polymer.

### 3.2. Theoretical Evaluation of Drug-Polymer Miscibility

#### 3.2.1. Solubility Parameters

The solubility parameter is a measure of cohesive energy density (CED: the cohesive energy per unit volume) of a material. The cohesive energy represents the total attractive forces within a condensed state material and can be defined as the quantity of energy needed to separate the atoms/molecules of a solid or liquid to a distance where the atoms or molecules possess no potential energy, that is, no interactions occur between atoms and molecules [30]. Consequently, solubility parameters have been used to predict the solubility/miscibility of one component into/with another component [42]. For this study, the solubility parameters were calculated using the Van Krevelen method [43], rather than the Fedors method [44,45], since the former considers hydrogen bonding. The Van Krevelen method provides:

(2) $$\delta =\sqrt{{\delta_{d}}^{2}+{\delta_{p}}^{2}+{\delta_{h}}^{2}}$$

$$\mathrm{where}\;\delta_{d}=\frac{\sum F_{di}}{V}\delta_{p}=\frac{\sqrt{\sum {F_{\mathrm{pi}}}^{2}}}{V}\delta_{h}=\sqrt{\frac{\sum E_{hi}}{V}}$$

where δ is the total solubility parameter; δ_d_, the contribution from dispersion forces; δ_p_, the contribution from polar forces; δ_h_, the contribution of hydrogen bonding; *F_di_*, the molar attraction constant due to dispersion component; *F_pi_*, the molar attraction constant due to polar component; *E_hi_*, the hydrogen bonding energy; and *V*, the molar volume. For various groups, the values of *F_di_*, *F_pi_*, *E_hi_*, and *V* (molar volume) are given in the literature [43,45]. The solubility parameters of these five polymers were taken from our previous study [29] and the solubility parameters calculated for IB and PVP are in good agreement with the literature [46].

Compounds with similar values of δ are likely to be miscible because the energy required to break interactions within each component is balanced by the energy released by interaction between the components. Greenhalgh et al. [30] classified dispersions based on the difference between the solubility parameters of excipients and drugs (Δδ). The authors demonstrated that compounds with Δδ < 7.0 MPa^1/2^ are likely to be miscible. However, compounds with Δδ > 10.0 MPa^1/2^ are likely to be immiscible. The calculated solubility parameters for IB, PVP, PMOZ, PEOZ, PnPOZ, and PiPOZ are summarized in Table 2.

It can be seen from Table 2 that all the polymers are expected to be miscible with IB with Δδ values ranging from 3.1 to 6.9, except for PMOZ (Δδ = 7.6). The rank order values for Δδ miscibility (PiPOZ; PnPOZ; PEOZ; PVP) are inconsistent with their ability to disrupt ibuprofen crystallinity, which may be explained by confounding factors such as the stronger hydrogen bonding seen between PVP and IB, as suggested from the FTIR data (Table 1). Although widely used, this approach has limitations and tends to be most widely applicable for drug–polymer systems where Van der Waals interactions play a major role, whereas for drug–polymer mixtures forming highly directional interactions such as hydrogen bonds or long range forces such as ionic interactions, this method can yield erroneous results [1,47].

#### 3.2.2. Flory–Huggins Interaction Parameter

Flory–Huggins theory considers melting point depression as an indicator of miscibility. According to this [48], the relationship between the melting temperature of the pure drug (*T*_*m*_^0^) and the depressed melting point of the drug in the drug–polymer system (*T_m_*) can be described by the following equation [49,50,51]:

(3) $$\frac{1}{T_{m}}-\frac{1}{T_{m}^{0}}=-\frac{R}{\Delta H}\left(\ln\Phi +\left(1-\frac{1}{m}\right)\left(1-\Phi \right)+\chi {\left(1-\Phi \right)}^{2}\right)$$

where *R* is the gas constant (8.31 J/mol·K), Δ*H* is the heat of fusion of the pure drug, *Φ* is the volume fraction of the drug in the solid dispersion (i.e., drug loading), *m* is the volume ratio between polymer and drug, and *χ* is the drug–polymer interaction parameter representing the difference between the drug–polymer contact interaction and the average self-contact interactions of drug–drug and polymer–polymer [49]. A negative *χ* value indicates that the interaction between a polymer and a drug is stronger than the attraction within polymer–polymer and drug–drug pairs. More negative values of *χ* indicate better affinity between the polymer and the drug and, for example, could be caused by hydrogen bonding between the drug and the polymer. Positive *χ* values indicate that drug molecules and polymer segments have stronger affinity to interact with those of their own kind rather than interacting with each other [50].

Given that all the polymer–IB solid dispersion systems showed depressed drug melting points at (polymer)/(drug) = 0.3:1 mol, the *χ* values of these dispersions were calculated and are listed in Table 3. Again, the PMOZ-IB system could not be investigated by this method.

As can be seen, the drug–polymer interaction parameters are all negative and broadly similar, ranging from −3.85 for PEOZ-IB to −3.32 for PnPOZ-IB. Interestingly, the Flory–Huggins approach suggests a rank order of (greatest interactions to least) of PEOZ > PVP > PiPOZ > PnPOZ, whereas the rank order of solubility parameter miscibility was PiPOZ > PnPOZ > PEOZ > PVP. Clearly, both approaches provide approximations (almost a “yes/no” guide), rather than a predictive ability to develop the optimal solid dispersion, since other factors influence the polymer’s ability to disrupt the drug’s crystallinity.

The X-ray, thermal, and infrared studies showed that PMOZ has a lower propensity to disorder ibuprofen than the other polymers. Its solubility parameter difference to ibuprofen (Δδ) was 7.6 MPa^1/2^, and thus beyond the notional value of 7 for miscibility but close to the borderline value 6.9 MPa^1/2^ calculated for PVP, which has the greatest tendency to disorder the drug. An alternative explanation is that the hydrophobic–hydrophilic balance (HHB) value for PMOZ (3.95) demonstrates that it is highly hydrophilic, and so the hydrophobic IB molecules will be less likely to molecularly disperse into the hydrophilic domains of PMOZ, consistent with our earlier findings on the non-hydrogen bonding dispersions with haloperidol where, again, PMOZ showed reduced interactions compared with the more hydrophobic carriers [29]. The importance of polymer hydrophobicity for crystal growth inhibitors has previously been reported [16].

### 3.3. In Vitro Dissolution Studies

**Figure 5:**
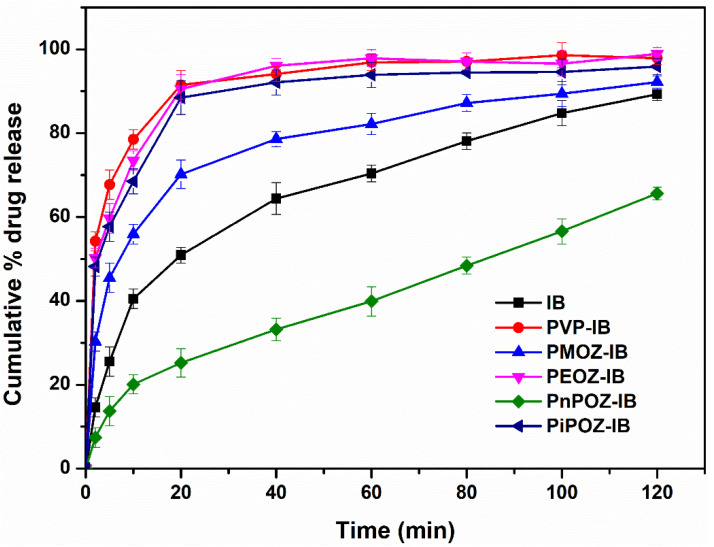
Dissolution profiles of pure IB and from different polymer–IB solid dispersions ((polymer repeat unit)/(drug) = 1:1 mol/mol). Cumulative % drug release with standard error of mean has been plotted against time.

The dissolution profiles of IB and polymer–IB (all 1:1 mol/mol) solid disperisons are shown in Figure 5. The dissolution of pure IB within 60 min was below 70%, with ~50% released in the first 20 min. As expected from the crystallinity data (Figure 4), dissolution was rapid from solid dispersions with PVP, PEOZ, and PiPOZ, where over 80% of the drug was released in the first 20 min. Drug release from solid dispersion with PMOZ was slower compared to PVP, PEOZ, and PiPOZ, but faster than IB alone, with ~70% of the drug released in 20 min, consistent with the analytical and theoretical discussions above. Despite the reduction of drug crystallinity and the system being essentially amorphous as determined by XRD and DSC, dispersions formed with PnPOZ showed slower dissolution than pure crystalline IB, with less than 30% released in 20 min. This result is consistent with our previous study [29] and can be explained by this polymer’s lower critical solution temperature (LCST) of ~25 °C [53,54], which is much lower than the temperature used in the dissolution studies (37 °C). Under these conditions, PnPOZ remains insoluble in the dissolution medium, which limits drug release from these solid dispersions. Further detailed dissolution studies of these formulations will be of interest in the future, for example, in assessing release below 25 °C and evaluating the extent of IB supersaturation on the evolution of kinetic solubility profiles [55].

## 4. Conclusions

Solid dispersions of IB were prepared using the poly(2-oxazolines) and PVP. Physical characterization of the dispersions showed that the polymers were able to disrupt the ibuprofen crystallinity, forming apparently amorphous dispersion at 1:1 mole ratios, and that hydrogen bonding was the prime mechanism for the interaction; however, the interactions between PMOZ and ibuprofen were more complex and hydrogen bonding was less prominent. The theoretical approach using the differences in solubility parameters between the drug and carrier or calculating the Flory–Huggins interaction parameters suggested compatibility between the drug and carriers, but the rank order of the predicted interactions varied between the two approaches. The purpose of generating solid dispersions is to enhance the dissolution rate of a poorly water soluble drug, and our studies demonstrated that the dispersions were able to significantly increase ibuprofen dissolution. However, our studies also show that other factors can significantly impact the performance of a solid dispersion. Physical characterization, for example, XRD showing that the drug is amorphous, can be assumed to result in enhanced dissolution. However, we show that not only is the crystallinity of a drug affected by dispersion, but so too is the structure of a semi-crystalline polymer (PiPOZ). The hydrophilicity of a carrier may reduce interactions with a hydrophobic drug, and so HHB may be an additional factor. Furthermore, the solution behavior of the carrier can also influence performance; physical characterization and theoretical models implied that dispersions with PnPOZ would be as effective as the other carriers, but the lower critical solution temperature (~25 °C) meant that this amorphous dispersion performed worse than the ibuprofen alone in the dissolution studies. Thus, both physical interactions, such as hydrogen bonding, and polymer properties, such as hydrophobicity, need to be considered when selecting carriers for solid dispersions.

## Figures and Tables

**Table 1:** The red shift of the carbonyl stretching mode from the carboxylic acid of IB at 1710 cm^−1^ in (polymer)/(IB) = 0.3 mol and 1:1 mol solid dispersions.

Polymer–Drug	Wavenumbers (cm^−1^)			
	0.3:1 mol	Red Shift	1:1 mol	Red Shift
PVP-IB	1727	17	1727	17
PMOZ-IB	1710	0	1713	3
PEOZ-IB	1711	1	1727	17
PnPOZ-IB	1713	3	1727	17
PiPOZ-IB	1712	2	1727	17

**Table 2:** Solubility parameters of drug and polymers.

Drug and Polymers	Solubility Parameters (δ) (MPa^1/2^)		Group Classification
	Van Krevelen Method	Δδ	
IB	19.4		
PVP	26.3	6.9	Miscible
PMOZ	27.0	7.6	Not miscible
PEOZ	24.5	5.1	Miscible
PnPOZ	22.9	3.5	Miscible
PiPOZ	22.5	3.1	Miscible

**Table 3:** Flory–Huggins interaction parameters of polymer–IB solid dispersion systems at the molar ratio of 0.3:1.

Polymer–Drug	V_Polymer Repeat Unit_ *^a^* (cm^3^/mol)	V _Polymer_ *^b^* (cm^3^/mol)	V_Drug_ *^c^* (cm^3^/mol)	M *^d^*	T_m_ (°C)	*χ*
PVP-IB	80.0	40,000	195.5	204.60	73.27	−3.71
PEOZ-IB	74.1	37,050		189.51	73.80	−3.85
PnPOZ-IB	90.2	45,100		230.69	73.50	−3.32
PiPOZ-IB	90.5	45,250		231.46	73.34	−3.52

*a* is the molecular volume of polymer repeating unit, calculated from the literature [43,45]. *b* is the molecular volume of polymer, calculated by multiplying V_polymer repeat unit_ by the repeat unit number. *c* is the molecular volume of IB, calculated from the literature [43,45], and is in agreement with the value taken from [52]. *d* is the volume ratio between the polymer and the drug.

## Data Availability

Data is contained within the article and in supplementary material.

## Supplementary Materials

The following are available online at https://www.mdpi.com/article/10.3390/pharmaceutics13050659/s1, Figure S1. FTIR of ibuprofen. Figure S2: FTIR full spectra of PVP-IB SDs (**a**), PEOZ-IB SDs (**b**), PnPOZ-IB SDs (**c**), PiPOZ-IB SDs (**d**) and PMOZ-IB SDs (**e**) Figure S3: FTIR spectra of PVP-IB SDs and POZ-IB SDs in the range of 1400~900 cm^−1^. The peaks (marked with an arrow) are attributed to C-N mode. Table S1: FTIR spectral data of ibuprofen (s—strong; w—weak; sym—symmetrical; asym—asymmetrical; str—stretching; m—medium; vs—very strong; vw—very weak.).
