# Supplementary material for: Mutual Effects of Hydrogen Bonding and Polymer Hydrophobicity on Ibuprofen Crystal Inhibition in Solid Dispersions with Poly(N-vinyl pyrrolidone) and Poly(2-oxazolines)
Source: Pharmaceutics. 2021 May 4;13(5):659. doi: 10.3390/pharmaceutics13050659 (PMC8148000; doi:10.3390/pharmaceutics13050659)
Supplement: Supplementary file 1 [file pharmaceutics-13-00659-s001.zip › pharmaceutics-1163171-supplementary.pdf]

pharmaceutics

Supplementary Materials: Mutual Effects of Hydrogen Bonding and Polymer Hydrophobicity on Ibuprofen Crystal Inhibition in Solid Dispersions with Poly(*N*-vinyl pyrrolidone) and Poly(2-oxazolines)

Xiaoning Shan, Maryam A. Moghul, Adrian C. Williams and Vitaliy V. Khutoryanskiy

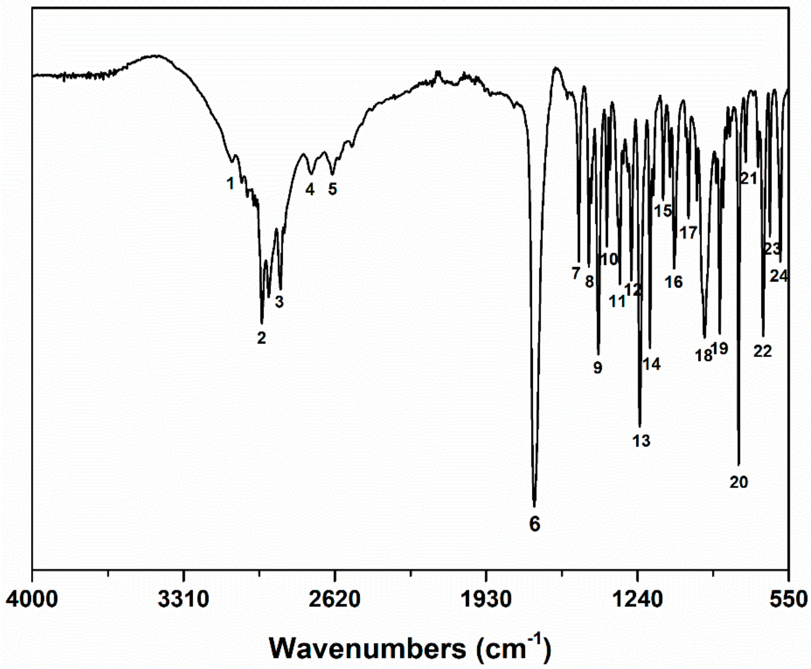

Figure S1. FTIR of ibuprofen.

(a)

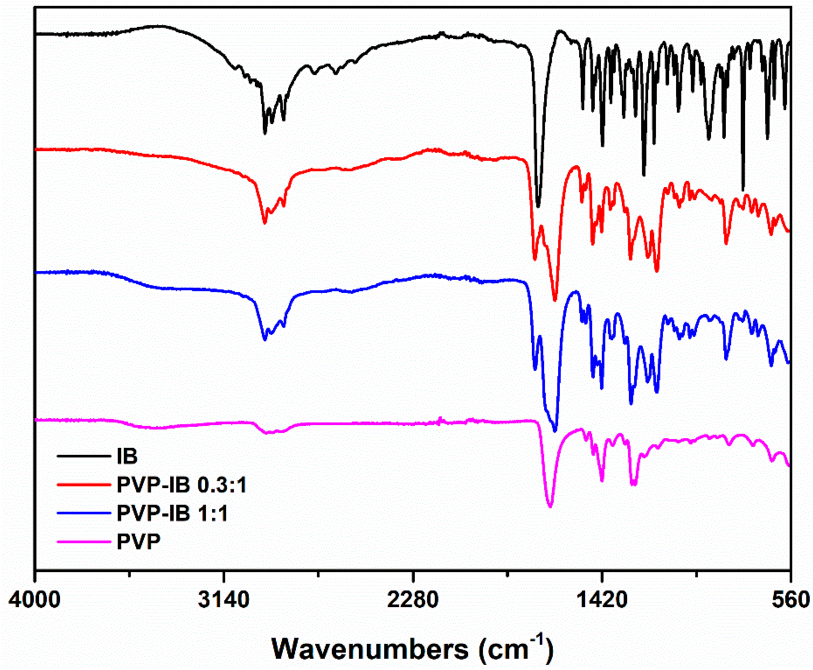
IB
PVP-IB 0.3:1
PVP-IB 1:1
PVP
4000
3140
2280
1420
560
Wavenumbers (cm-1)

(b)

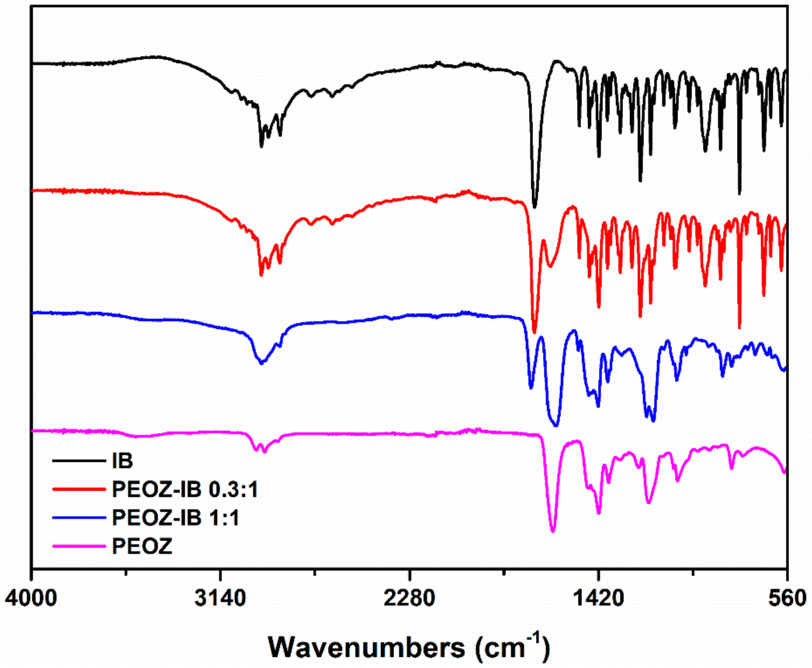
IB
PEOZ-IB 0.3:1
PEOZ-IB 1:1
PEOZ
4000
3140
2280
1420
560
Wavenumbers (cm-1)

(c)

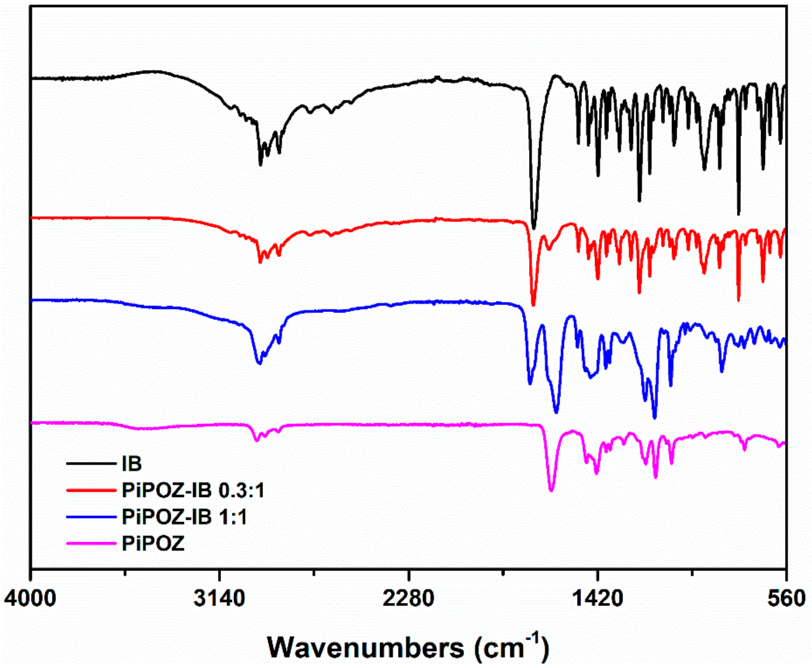
IB
PiPOZ-IB 0.3:1
PiPOZ-IB 1:1
PiPOZ
4000
3140
2280
1420
560
Wavenumbers (cm^{-1})

(d)

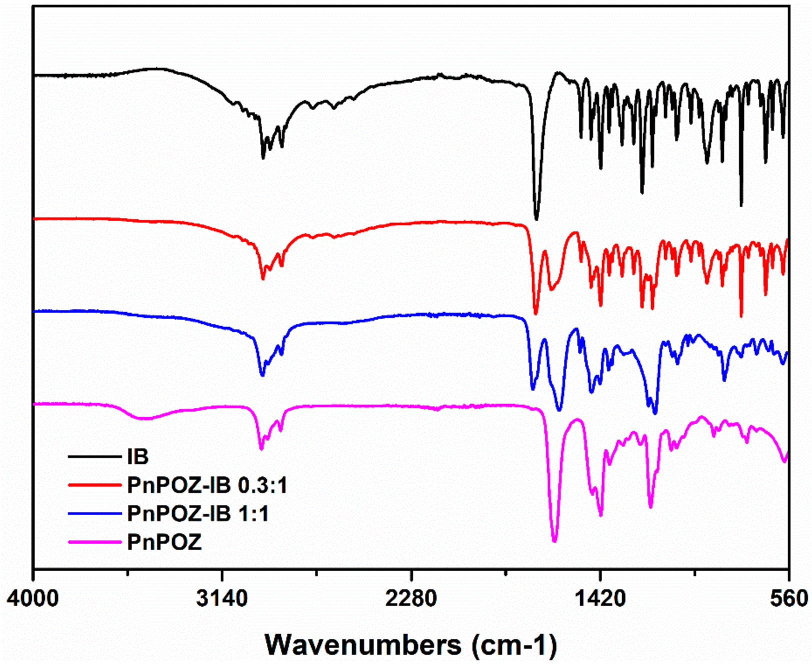
IB
PnPOZ-IB 0.3:1
PnPOZ-IB 1:1
PnPOZ
4000
3140
2280
1420
560
Wavenumbers (cm-1)

(e)

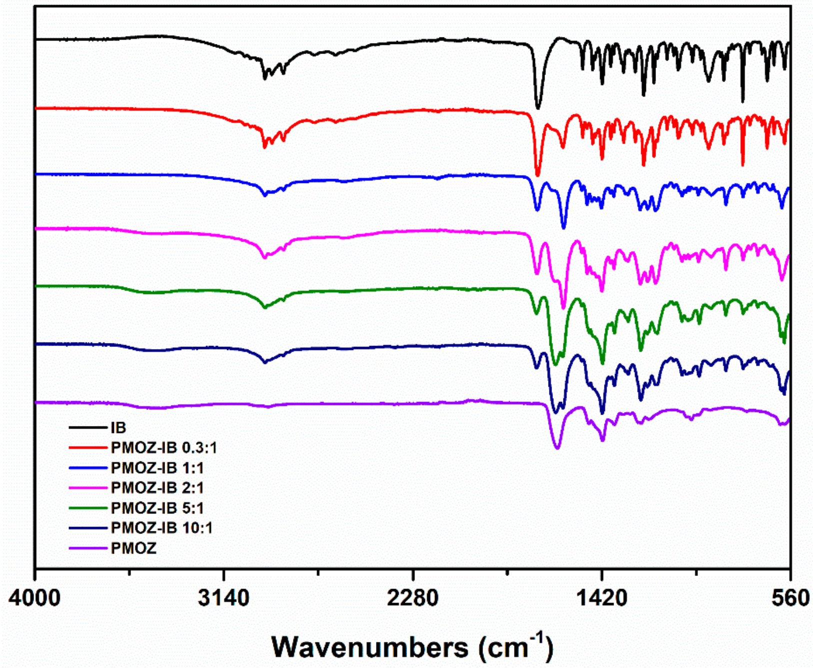

Figure S2. FTIR full spectra of PVP-IB SDs (**a**), PEOZ-IB SDs (**b**), PnPOZ-IB SDs (**c**), PiPOZ-IB SDs (**d**) and PMOZ-IB SDs (**e**).

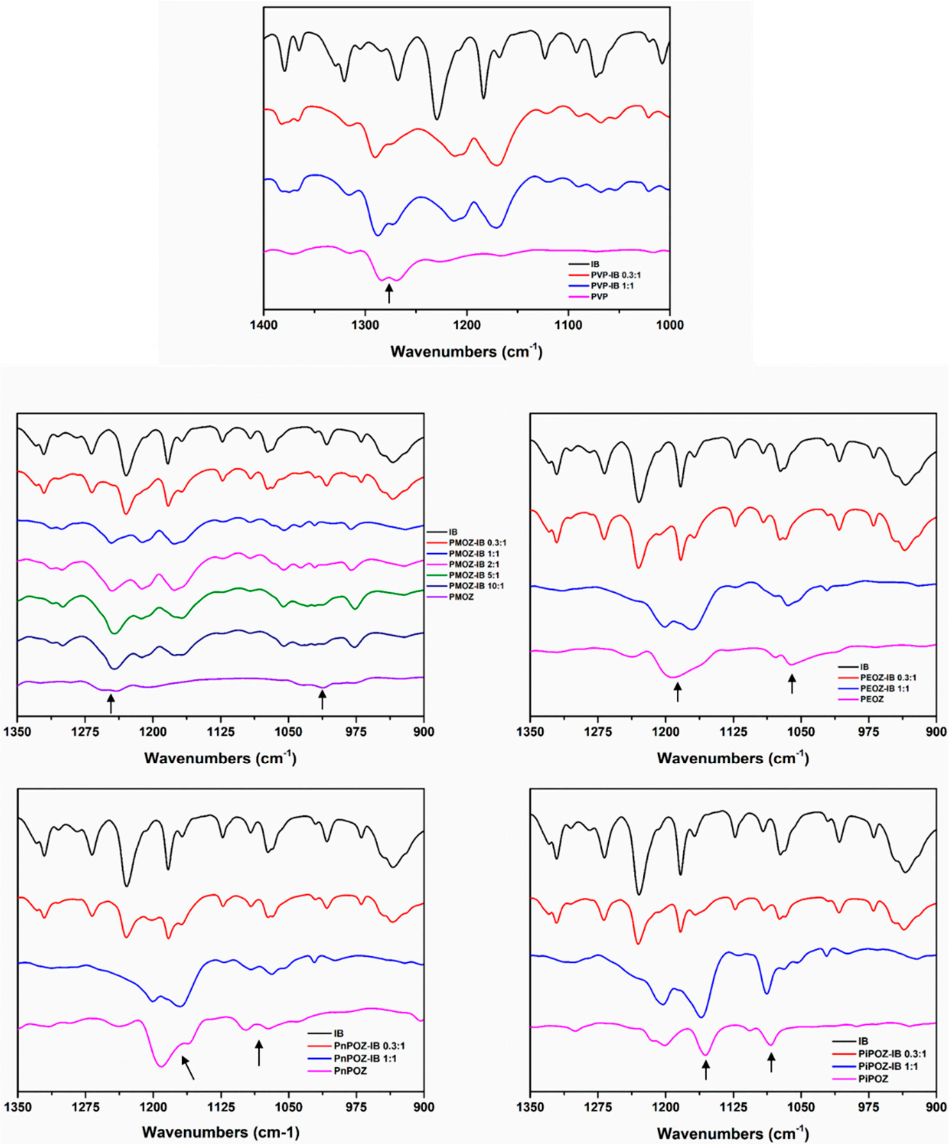

Figure S3. FTIR spectra of PVP-IB SDs and POZ-IB SDs in the range of 1400～900 cm^{-1}. The peaks (marked with an arrow) are attributed to C–N mode.

Table S1. FTIR spectral data of ibuprofen (s- strong; w- weak; sym-symmetrical; asym-asymmetrical; str-stretching; m-medium; vs- very strong; vw – very weak.).

Band	Wavenumbers (cm^{-1})	Assignments
1	3088 m	CH_2 asym str
2	2955 vs	CH_3 asym str
3	2869 m	CH_2 sym str
4	2725 m	O-H…O valence str
5	2633 m	O-H…O valence str
6	1710 vs	C=O str
7	1507 s	aromatic C=C str
8	1462 s	CH_3 asym deformation, CH_2 scissoring
9	1416 s	CH-CO deformation
10	1380 s	CH_3 sym str
11	1320 s	OH in plane deformation
12	1268 s	=C–H in plane deformation
13	1230 vs	C…C str
14	1183 s	C–O str

15	1123 w	=C–H in plane deformation
16	1071 m	=C–H in plane deformation
17	1008 m	C–H in plane deformation
18	936 s	CH_3 rocking vibration
19	866 s	C–H out of plane vibration
20	779 s	CH_2 rocking
21	746 w	C=C ring str, C...C skeletal vibration
22	668 s	C–H out of plane deformation
23	636 w	C–H in plane ring deformation
24	588 m	C...C deformation
